# Very high frequency ultrasonographic characteristics of poroma: a retrospective observational study

**DOI:** 10.1186/s12880-026-02484-z

**Published:** 2026-06-04

**Authors:** Bin Dong, Hongsheng Xia, Yan Xia, Li Chai, Bo Xie, Ying Liu

**Affiliations:** 1 Department of Ultrasound, Hangzhou Third People’s Hospital, Hangzhou Third Hospital Affiliated to Zhejiang Chinese Medical University, Hangzhou, China; 2 Department of Dermatology, Hangzhou Third People’s Hospital, Hangzhou Third Hospital Affiliated to Zhejiang Chinese Medical University, West Lake Rd 38, Hangzhou, 310009 China; 3 Cancer Center, Department of Ultrasound Medicine, Zhejiang Provincial People’s Hospital(Affiliated People’s Hospital), Hangzhou Medical College, No. 158 Shangtang Road, Hangzhou, Zhejiang 310014 China

**Keywords:** Poroma, Eccrine poroma, Very high frequency, Ultrasound, Diagnosis

## Abstract

**Background:**

Poroma is a rare benign skin tumor, diagnostic challenges of which require advanced imaging. Herein we investigate the ultrasonographic manifestations and clinical value of the very high frequency(VHF) ultrasound in the auxiliary diagnosis of Poroma.

**Methods:**

Clinical data of 24 cases pathologically diagnosed as poroma were retrospectively analyzed by means of high-resolution VHF ultrasound, including size, location, shape, margin, boundary, internal echo and blood flow patterns.

**Results:**

VHF ultrasound examination shows that most poroma are located in the epidermis and dermis. The maximum diameter was 2.6–30 (11.86 ± 6.31) mm. The tumor has clear borders, and the echo intensity of most lesions(22/24) was heterogeneous hypoechoic. Most lesions(18/24) showed small duct-like anechoic areas, 6 lesions(6/24) were detected with punctate calcification. In terms of blood flow, most lesions(18/24) showed abundant blood flow signals, which were dendritic.

**Conclusions:**

The VHF ultrasound lacks specificity in diagnosing poroma and currently makes accurate qualitative diagnosis difficult, but it holds significant value for assessment. It can clearly define the lesion’s location, layers, number, size, boundaries, relationship to surrounding structures, and internal blood supply. This provides an objective basis for selecting the next diagnostic or treatment plan, and it can also serve as an evaluation. method for postoperative follow-up.

## Background

Poroma is a rare benign tumor of cutaneous adnexal. Eccrine and apocrine tumors account for only 1% of all primary skin diseases, and of these lesions, poroma accounts for approximately 10%. It usually occurs in middleaged and elderly people [1]. There is no predilection for gender. It occurs mostly on the soles or palms, but can occur on any other skin site [2]. Poromas are defined into four subtypes: eccrine poroma (EP), hidracanthoma simplex, dermal duct tumor, and poroid hidradenoma [1]. The latest World Health Organization (WHO) classification defines these four lesions as poromas [3, 4]. Poroma usually manifests as a single, slowly growing papule, plaque, nodule. The surface can be smooth or warty [5]. The most poroma are skin-colored, and only about 17% are pigmented (from red to brown or blue) [6]. It is usually asymptomatic, with only mild itching or pain in a few patients. The pathogenesis of poroma is still unclear, recent studies have exhibited that gene fusions (such as YAP/TAZ, YAP1-MAML2, YAP1-NUTM1, etc.) play important roles in the incidence of poroma [1].

Imaging modalities for characterizing poromas include dermatologic ultrasound, dermoscopy [7], reflectance confocal microscopy (RCM) [8], and magnetic resonance imaging (MRI) [9]. Dermoscopy is commonly used for poroma evaluation, with eccrine poroma (EP) typically demonstrating dermoscopic features such as white interlaced areas surrounding vessels, structureless yellow areas, pink globules, and ill-defined vascular patterns. However, EP acts as a significant “mimicker” under dermoscopy, making differential diagnosis challenging [7]. Consequently, RCM features of EP have been investigated. Studies by Francesca Di Tullio et al. indicate that RCM provides certain diagnostic characteristics that can improve diagnostic accuracy. Beyond dermoscopy and RCM, Kawaguchi M et al. described MRI features through case reports [9]. As poromas share features with both benign and malignant skin tumors—such as seborrheic keratosis, squamous cell carcinoma, and basal cell carcinoma—histopathological examination remains essential for definitive diagnosis [1].

Ultrasound stands as a pivotal imaging technique in dermatology, offering advantages of accessibility and cost-effectiveness, making it a primary diagnostic tool for skin diseases. In recent years, advancements in high-frequency ultrasound technology have enabled the clinical use of probes exceeding 20 MHz, substantially enhancing resolution to visualize each skin layer distinctly. The frequency range of 30–75 MHz is defined as ultra-high-frequency (UHF), while 20–30 MHz corresponds to very-high-frequency (VHF) ultrasound [10] which allow displaying the skin layers and their abnormalities with sufficiently high-quality images and enabling differentiating structures of approximately 100 μm on the beam axis [11]. VHF ultrasound allows precise localization of the anatomical layers involved by lesions and provides detailed assessment of lesion size, internal echogenicity, vascular characteristics, and relationship to adjacent tissues. This capability guides clinical management and serves for postoperative follow-up evaluations. This study analyzed very-high-frequency (VHF) ultrasound images of 24 histopathologically confirmed poroma cases, summarizing their characteristics to offer a supplementary approach for clinical diagnosis.

## Methods

### Study Population

The data of 24 poroma patients admitted to Hangzhou Third People’s Hospital from June 2019 to June 2024 were collected. All of the patients were pathologically confirmed as poroma. Among all 24 cases, there were 20 patients with EP, 1 with hidracanthoma simplex, 2 with poroid hidradenoma, and 1 with dermal duct tumor.

### Image instruments and analysis process

All patients were examined with Color Doppler Ultrasound Diagnostic Equipment (Esaote MyLab™ One, Italy, 22 MHz probe). The VHF ultrasonographic features of 24 lesions were analyzed retrospectively, including size, location, skin layers involved, shape, margin, boundary, interior echo, blood supply. Above analysis was performed by two senior physicians who had more than 5 years of experience in dermatologic ultrasonographic medicine and finished the work independently of each other. And the two senior physicians were blinded to histopathological results. A third would join the analysis if there was no consensus. Kappa analysis was applied and features with poor consistency were eliminated.

## Results

### General conditions / clinical features

**Fig. 1:**
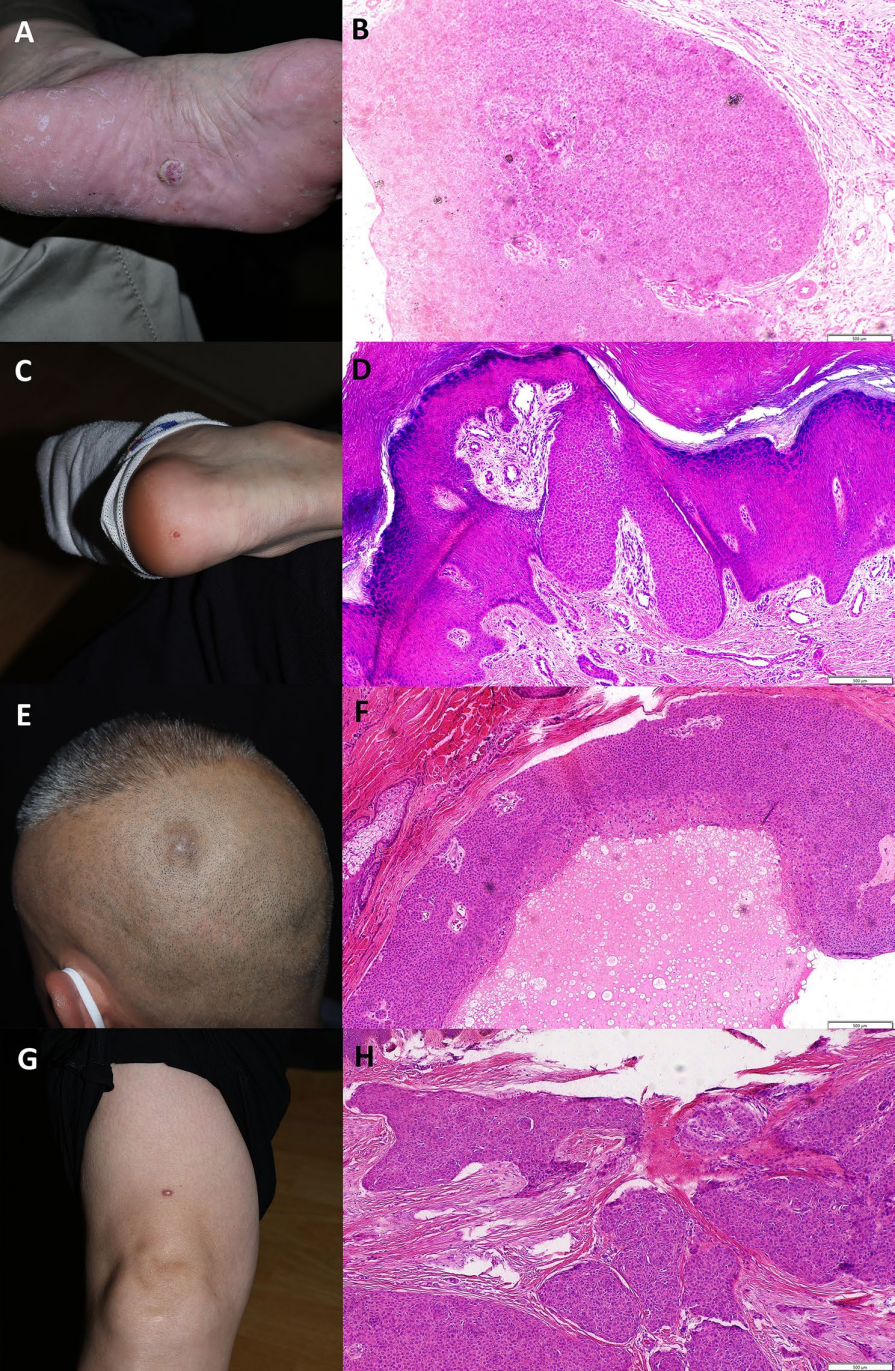
Clinical and pathological images of the four subtypes of poroma. **A**, **C**, **E**, **G**: clinical images of eccrine poroma, hidracanthoma simplex, poroid hidradenoma, and dermal duct tumor (corresponding to #11, #21, #22, and #24, in Table 1); **B**, **D**, **F**, **H**: pathological images corresponding to **A**, **C**, **E**, **G**

The general conditions of 24 poroma patients (13 male, 11 female; age range, 18–69 years; mean age, 48.25 years) is shown in Table 1. All 24 patients were confirmed by pathological examination, including 20 cases of EP (Table 1 #1–20), 1 case of hidracanthoma simplex (Table 1 #21), 2 cases of poroid hidradenoma (Table 1 #22–23) and 1 case of dermal duct tumor (Table 1 #24). Representative clinical and pathological images could be found in Fig. 1A-H. The most common site of lesion is the sole (13/24). Most poromas (15/24) projected above the skin surface as polypoid lesions. These 15 polypoid lesions included 10 broad-based and 5 narrow-based growths.

**Table 1:** VHF ultrasound characteristics of 24 cases of poroma

Case	Age / Sex	Site	Size (mm)	Pedicle	Subtypes	Internal Echo Features	Skin Layers Involved	Blood Vessels ^a^	SLTS	CA	Enhanced Rear Echo
# 1	30 / M	Temporal	30 × 21		EP	Uneven hypoechoic	Dermis & ST	Ⅲ ^b^		Yes	Yes
# 2	65 / F	Sole	9.8 × 3.8		EP	Uneven hypoechoic	Epidermis & Dermis	Ⅲ ^b^	Yes	Yes	Yes
# 3	61 / F	Sole	18.8 × 5.1	Wide	EP	Uneven hypoechoic	Epidermis & Dermis	Ⅲ ^b^	Yes	Yes	Yes
# 4	48 / M	Sole	12.4 × 4.9	Wide	EP	Uneven hypoechoic	Epidermis & Dermis	Ⅲ ^b^	Yes		Yes
# 5	55 / F	Sole	4.7 × 1.5		EP	Uneven hypoechoic	Epidermis & Dermis	Ⅲ ^b^	Yes		Yes
# 6	68 / F	Sole	2.6 × 1.1		EP	Uneven hypoechoic	Epidermis & Dermis	Ⅱ			Yes
# 7	43 / F	Hip	9.2 × 4.0	Narrow	EP	Uneven hypoechoic	Epidermis & Dermis	Ⅲ ^b^	Yes		
# 8	27 / M	Limb	9.5 × 3.2	Wide	EP	Uneven hypoechoic	Epidermis & Dermis	Ⅲ ^b^	Yes	Yes	
# 9	65 / M	Sole	11.0 × 2.9	Wide	EP	Uneven hypoechoic	Epidermis & Dermis	Ⅲ ^b^	Yes		
# 10	69 / M	Sole	9.6 × 2.4	Wide	EP	Uneven hypoechoic	Epidermis & Dermis	Ⅲ ^b^	Yes		
# 11	68 / M	Sole	10.8 × 4.5	Narrow	EP	Uneven hypoechoic	Epidermis & Dermis	Ⅲ ^b^	Yes		
# 12	43 / M	Ankle	10.8 × 3.5	Wide	EP	Uneven hypoechoic	Epidermis & Dermis	Ⅲ ^b^	Yes		
# 13	65 / F	Hip	18.0 × 12.5	Narrow	EP	Uneven hypoechoic	Epidermis & Dermis	Ⅲ ^b^	Yes		
# 14	33 / F	Scalp	9.3 × 4.9	Narrow	EP	Uneven hypoechoic	Epidermis & Dermis	Ⅲ ^b^	Yes		
# 15	34 / F	Sole	11.1 × 2.6	Wide	EP	Uneven hypoechoic	Epidermis & Dermis	Ⅲ ^b^	Yes		Yes
# 16	18 / M	Occipital	22.6 × 16.0		EP	Uneven hypoechoic	Dermis & ST	Ⅱ	Yes		Yes
# 17	28 / F	Sole	19.2 × 10.8		EP	Uneven hypoechoic	Dermis & ST	Ⅱ	Yes		Yes
# 18	68 / M	Sole	9.5 × 3.6	Wide	EP	Uneven hypoechoic	Dermis & ST	Ⅲ ^b^	Yes		
# 19	32 / M	Sole	10.2 × 3.4	Wide	EP	Uneven hypoechoic	Epidermis & Dermis	Ⅲ ^b^	Yes		
# 20	30 / M	Palm	9.9 × 3.2	Wide	EP	Uneven hypoechoic	Epidermis & Dermis	Ⅲ ^b^	Yes		Yes
# 21	29 / M	Sole	3.7 × 1.9	Narrow	HS	Uneven hypoechoic	Epidermis	Ⅱ			Yes
# 22	50 / M	Scalp	12.7 × 6.2		PH	Liquid-based mixed echo	Dermis & ST	0			
# 23	68 / F	Back	15.6 × 7.4		PH	Liquid-based mixed echo	Dermis & ST	0			
# 24	61 / F	Knee	3.6 × 1.7		DDT	Uneven hypoechoic	Epidermis & Dermis	Ⅲ		Yes	

a: Blood flow with Adler’s method. Grade 0: No blood signal. Grade I: A small amount of blood signal, 1–2 point-like blood flow (diameter <1 mm). Grade II: Moderate blood flow, one major vessel (exceeds the radius of the lesion) or 2–3 small vessels. Grade III: Abundant blood flow is (≥ 4 vessels or the vessels are interconnected into a network. b: Dendritic blood vessels. ST: Subcutaneous tissue. SLTS: Small liquid tubular structure. CA: Calcification. EP: eccrine poroma. HS: hidracanthoma simplex. PH: poroid hidradenoma. DDT: dermal duct tumor

### VHF ultrasound characteristics

**Fig. 2:**
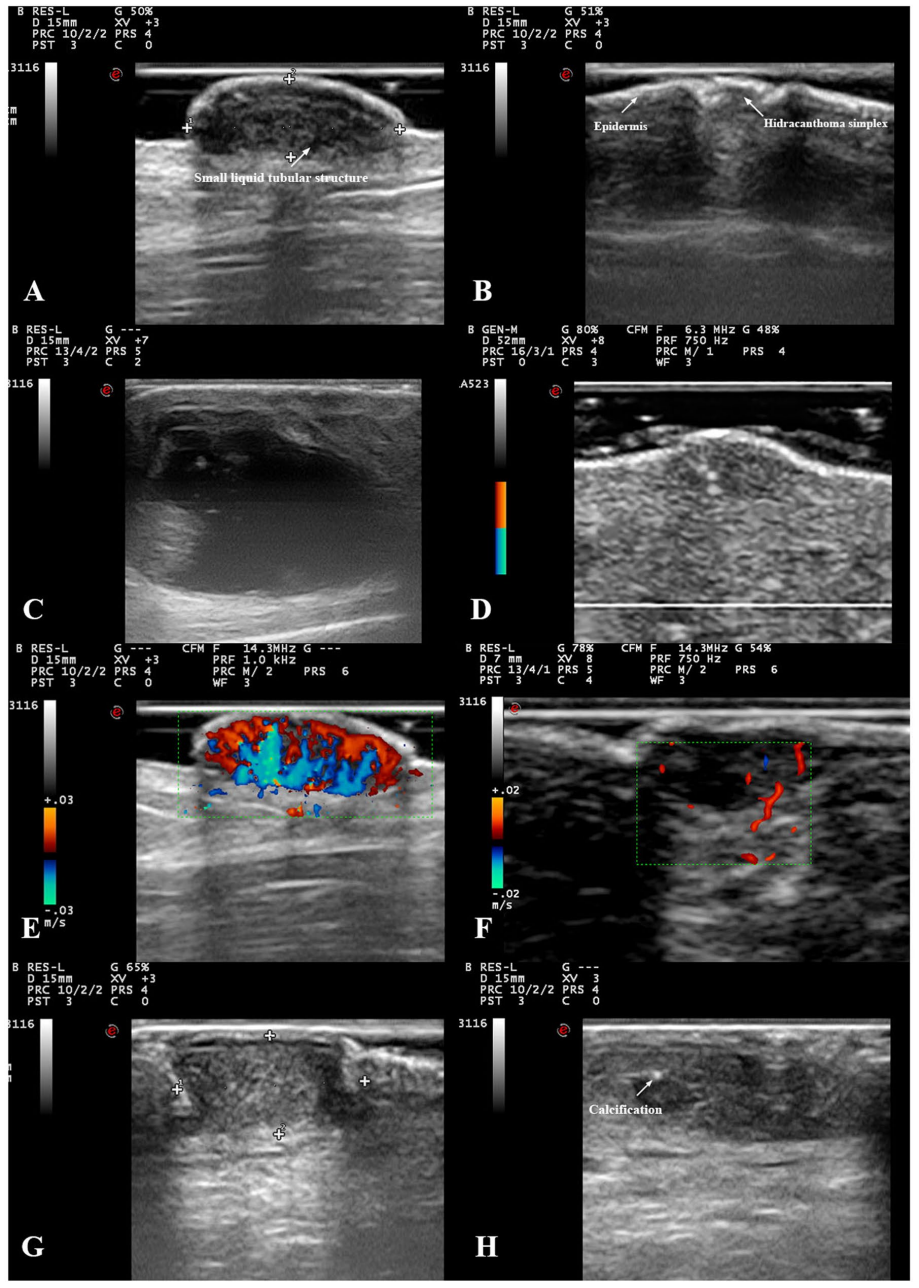
The VHF ultrasound characteristics of 24 poromas. **A**: eccrine poroma involving the epidermis and dermis layers, with heterogeneous hypoechoic and small liquid tubular structure; **B**: hidracanthoma simplex only involving the epidermis; **C**: poroid hidradenoma with liquid-based mixed echo; **D**: dermal duct tumor; **E**: grade Ⅲ of blood flow with Adler’s method, dendritic blood vessels; **F**: grade Ⅱ of blood flow; **G**: enhanced rear echo; **H**: calcification

The VHF ultrasound characteristics of 24 poromas were shown in Table 1. The maximum diameter was 2.6–30 (11.86 ± 6.31) mm. There were 18 cases where the lesions were located in both epidermis and dermis, 5 cases were located in both dermis and subcutaneous tissue, and 1 case was located only in the epidermis (hidracanthoma simplex, Fig. 2B). The lesions of 22 patients showed uneven hypoecho (Fig. 2A). Only 2 cases showed mixed echoes, mainly cystic echoes (Fig. 2C), and both were pathologically classified as poroid hidradenoma. Among the 20 cases of EP, 18 cases showed small internal duct-like anechoic fluid echogenic areas in the lesions (Fig. 2A). This feature was not seen in the other three pathological types. In addition, there were 5 cases with calcification (Fig. 2H) inside the lesions, and 11 cases with enhanced posterior echoes (Fig. 2G). As for the blood flow within the lesion, it was based on the Adler grading method. Among the 20 EP patients, 17 showed grade III (rich blood flow signal, PSV 22.12 ± 2.64 cm/s, RI 0.58 ± 0.07) and dendritic vessels (Fig. 2E). The remaining 3 cases of EP showed grade II (moderate blood flow signal, PSV 14.57 ± 3.42 cm/s, RI 0.61 ± 0.08) (Fig. 2F). Different from EP, both cases of poroid hidradenoma were grade 0 (no blood flow signal was seen). In dermal duct tumor (Fig. 2D), although tertiary blood flow can be seen, dendritic blood vessels are not seen. A case of hidracanthoma simplex shows secondary blood flow.

## Discussion

Poroma is a pretender in terms of clinical manifestations and dermoscopic characteristics, coupled with its low incidence rate. Dermatologists may have insufficient knowledge of this disease [8]. Poroma can occur on any part of the body. The most predilection sites were lower limbs, of which feet were the most common. In this case series, the foot was the most frequent location, followed by the head, trunk, upper limbs, and knee. Notably, two cases occurred on the buttocks, a relatively uncommon location in the currently published literature [12]. Most poromas in this group are painless and slow-growing with a maximum diameter of 3 cm and a longest history up to 10 years. The appearance of a typical poroma is characterized by solitary, red or pigmented nodules. In this study, more than half of the lesions exhibited a polypoid morphology, with some being broad-based and others narrow-based. This characteristic was not described in previous reports, possibly due to the single pathological type and limited sample size in prior studies. Patients are prone to bleeding due to telangiectatic hyperplasia within the lesion, [7] a finding consistent with our analysis, as half of the lesions in this case series were associated with ulceration.

Poromas are rare and clinically challenging to diagnose due to insufficient awareness, leading to a high misdiagnosis rate. In recent years, the development of high-frequency ultrasound technology has enriched the auxiliary diagnostic methods for skin diseases. VHF ultrasound offers significantly greater value than traditional ultrasound, with markedly higher resolution. It can precisely locate the anatomical layers involved by the poroma and provide detailed assessment of the lesion’s size, internal echogenicity, vascularity, and relationship to adjacent tissues. This assists in guiding clinical diagnosis and treatment and serves as an important tool for postoperative follow-up evaluations. Because poromas are rare, there are currently very few published studies analyzing their ultrasonographic features, with the existing literature primarily consisting of case reports.

In previous studies [13–17] evaluating high-frequency ultrasound characteristics, poromas were most commonly found involving the epidermal and dermal layers. Larger lesions may extend into the subcutaneous tissue. They are typically solitary, well-demarcated lesions, exhibiting either a regular oval/round shape or an irregular lobulated configuration. Internally, they most commonly appear as solid hypoechoic masses, though they may also demonstrate mixed cystic-solid echogenicity. Some lesions show a layered echotexture pattern with hypoechoic areas alternating with patchy or short linear hyperechoic areas. Others may contain multiple punctate hyperechoic foci. Vascularity assessment typically reveals either abundant or minimal blood flow signals.

In this study, most poroma lesions were located within the epidermis and dermis. The second most common pattern involved simultaneous extension into both the dermis and subcutaneous layer, while a minority were confined solely to the epidermis (identified as hidroacanthoma simplex). Lesions typically exhibited well-defined borders, likely attributable to the proliferation of basaloid tumor cells that contrast distinctly with surrounding squamous epithelium.^2^ Most lesions demonstrated heterogeneously hypoechoic echogenicity, though some presented with mixed echogenicity featuring predominantly cystic components and displaying a “fluid-fluid level sign” – consistent with reported ultrasonographic characteristics of apocrine hidradenoma [18]. This variation may relate to pathological subtypes, as both such cases were classified as poroid hidradenoma, a subtype particularly prone to liquefactive necrosis. Posterior acoustic enhancement was observed in some lesions, potentially associated with focal ductal differentiation, cyst formation, or necrotic changes within the tumor. The internal echotexture of most lesions revealed small tubular anechoic areas, with punctate calcifications detected in others. These features may correspond to diffuse or focal calcification within the tumor’s ductal structures. Regarding vascularity, our findings align with previous studies reporting abundant blood flow signals in most lesions. Only two cases of poroid hidradenoma showed no detectable vascularity, correlating with the sparse vascularity within fibrous stroma characteristic of this subtype. We further observed that most lesions exhibited a consistent dendritic vascular pattern on VHF ultrasound, resembling the radial blood flow signals described in Zuo’s case report [16].

Due to the rarity of poromas, differentiating them from other benign and malignant skin tumors is challenging. However, understanding the sonographic characteristics of other cutaneous neoplasms aids in analyzing the features of poromas. Clinically, poromas may be misdiagnosed as various benign tumors of skin origin, such as seborrheic keratosis, acrochordon (skin tag), or pilomatricoma (calcifying epithelioma). Seborrheic keratosis lesions are confined to the epidermis with no significant dermal changes. The surface is typically hyperechoic, and Color Doppler Flow Imaging (CDFI) usually shows relatively abundant blood flow signals [19]. Acrochordons often present as round or oval, solid, exophytic masses with relatively homogeneous internal echogenicity. Internal vascularity is typically sparse, and a pedunculated structure connecting to the skin is characteristically visible [20]. Pilomatricomas usually appear as oval nodules in the deep dermis or subcutaneous tissue, predominantly hypoechoic, often containing scattered punctate or plaque-like hyperechoic foci (representing calcification) [21]. The main malignancies developing from dermal components are dermatofibrosarcoma protuberans (DFSP), which can grow into the subcutaneous tissue. They often have regular shapes, sometimes with small lobulations, mostly well-defined borders, and heterogeneous hypoechoic echogenicity. Posterior acoustic enhancement is common in most lesions, which typically show abundant internal vascularity with measurable low-to-intermediate resistance arterial spectra [22]. Malignant tumors of skin appendages, also arising in the dermal layer, include eccrine porocarcinoma, which is even rarer than poromas. According to previously reported cases, they tend to be well-demarcated with heterogeneous internal echogenicity, often containing punctate hyperechoic foci. CDFI demonstrates punctate or linear blood flow signals within and around the lesion [23]. Although the sonographic features of poromas lack high specificity, ultrasound can precisely localize the anatomical layers involved by the tumor. It also provides detailed characterization of the lesion’s internal echotexture, vascular features, and relationship to surrounding tissues, thereby providing valuable guidance for selecting appropriate clinical management strategies.

Limitations of this study include its single-center design and relatively small sample size, which preclude a comprehensive and systematic summary of the ultrasonographic characteristics of poromas. Future studies should expand the cohort to further investigate the sonographic features of poromas.Furthermore, there was insufficient utilization of advanced imaging technologies. Specifically, microvascular imaging techniques (e.g., Superb Microvascular Imaging/SMI) [24] offer greater clinical value than the Adler grading system for assessing vascular patterns in cutaneous lesions and additionally enable quantitative evaluation of blood flow.

## Conclusion

Our research contributes to the limited knowledge on this rare condition and highlights the potential of VHF ultrasound. The high resolution of VHF ultrasound allows for the detailed visualization of superficial lesions. VHF ultrasound demonstrates value in diagnosing poromas, though it currently lacks high specificity and cannot provide definitive qualitative diagnosis. Nevertheless, it can accurately delineate key lesion characteristics including location, tissue layer involvement, number, size, borders, relationship to adjacent structures, and internal vascular characteristics. This information provides an objective basis for selecting subsequent management strategies and serves as a valuable tool for postoperative follow-up assessments.

## Data Availability

The datasets generated during and/or analyzed during the current study are available from the corresponding author on reasonable request.

## Abbreviations

VHF: Very high frequency
EP: Eccrine poroma
WHO: World Health Organization
RCM: Reflectance confocal microscopy
MRI: Magnetic resonance imaging
UHF: Ultra-high-frequency
CDFI: Color Doppler Flow Imaging
DFSP: Dermatofibrosarcoma protuberans
SMI: Superb Microvascular Imaging
